# Research-ready data: the C-Surv data model

**DOI:** 10.1007/s10654-022-00916-y

**Published:** 2023-01-07

**Authors:** Sarah Bauermeister, Joshua R Bauermeister, Ruth Bridgman, Caterina Felici, Mark Newbury, Laura North, Christopher Orton, Emma Squires, Simon Thompson, Simon Young, John E Gallacher

**Affiliations:** 1 Department of Psychiatry, University of Oxford, Oxford, United Kingdom; 2 Swansea University Medical School, Swansea University, Swansea, United Kingdom

## Abstract

Research-ready data (data curated to a defined standard) increase scientific opportunity and rigour by integrating the data environment. The development of research platforms has highlighted the value of research-ready data, particularly for multi-cohort analyses. Following stakeholder consultation, a standard data model (C-Surv) optimised for data discovery, was developed using data from 5 population and clinical cohort studies. The model uses a four-tier nested structure based on 18 data themes selected according to user behaviour or technology. Standard variable naming conventions are applied to uniquely identify variables within the context of longitudinal studies. The data model was used to develop a harmonised dataset for 11 cohorts. This dataset populated the Cohort Explorer data discovery tool for assessing the feasibility of an analysis prior to making a data access request. Data preparation times were compared between cohort specific data models and C-Surv.

It was concluded that adopting a common data model as a data standard for the discovery and analysis of research cohort data offers multiple benefits.

**Supplementary Information:**

The online version contains supplementary material available at 10.1007/s10654-022-00916-y.

## Introduction

Research-ready data (data curated to a defined standard) offer many advantages. For data producers, defined data standards provide a template for data management. For multi-lateral collaborations, a defined standard obviates the need to integrate multiple bespoke data models. For third-party scientists, research-ready data remove the need to repeatedly and idiosyncratically curate data on a per-project basis. Overall, research-ready data make data more accessible and more convenient to use. They reduce the cost of data management, the cost of collaboration, and uncertainty. These outcomes deliver increased opportunity, pace, and rigour.

These advantages have been clearly demonstrated in the introduction of reference SNP cluster ID (rs) numbers in genetics, and the Neuroimaging Informatics Technology Initiative (NIfTI) and Digital Imaging and Communications in Medicine (DICOM) imaging formats. Data standards have also been developed for trials data: CDISC - Clinical Data Interchange Standards Consortium [1], electronic health records: SNOMED – Systematized Nomenclature of Medicine [2]; OHDSI – Observational Health Data Sciences and Informatics [3]; FIHR – Fast Healthcare Interoperability Resources [4] and Pangolin for genetic linkage in infectious disease [5] and HPO – Human Phenotype Ontology [6] for chronic disease. For population-based cohort studies, however, there are no established data standards for research-based phenotypes. Typically, cohort studies use data models that have evolved over time according to each project’s scientific priorities and resource constraints; using bespoke structures and labelling conventions, and varying in the quality of curation and documentation. Whilst retrofitting cohort data to models developed for other purposes is possible, they have structural and semantic complexity that is alien to the natural organisation of longitudinal research data.

The growing interest in multi-cohort analyses and third-party data access, as expressed through the growth of data access platforms, prompted the design of a data model optimised for use with population-based cohort data. Implicit in the development of a data model is the underlying ontology. This is the conceptual data space where all the facts (observations concerning the elements of the data) and relationships between facts (observations concerning the structure of the data), are defined. The expression of these rules is the data model. The data space need not be complex as its function is to simplify and standardise. A data model that simplifies addressing complex questions is useful. Ontologies are like maps: information is recorded and structured selectively according to purpose.

This paper describes the development of the C-Surv ontology and data model; developed for use in the Dementias Platform UK (DPUK) Data Portal [7]. Our objective was to design and implement a data model suitable for the discovery and selection of research cohort data using neurodegeneration as a use case. For further details of the Data Portal, go to DPUK Data Portal (https://portal.dementiasplatform.uk).

## Methods

### Landscape review

To define the problem more closely, a landscape review was conducted. The DPUK Cohort Directory (https://portal.dementiasplatform.uk/CohortDirectory) was used to sample current practice. For 45 collaborating cohorts, details of the data and metadata models were sought from documentation provided by the cohorts and from the literature.

### Stakeholder engagement

Stakeholder engagement evolved according to need rather than being formal qualitative studies. User needs were initially identified through two stakeholder workshops (one in-person and one virtual) comprising cohort research team members, ontologists, data scientists, and data managers. For the stakeholder workshops, the mission statement was to create simple data conventions that could be applied to multi-cohort, multi-modal, data. To provide context, epidemiologic population cohorts were used as use-cases. Initial solutions were then presented during site-visits to 20 DPUK collaborating cohorts and at four international conferences and workshops [8–11]; feedback being invited at each.

### Design considerations

Design criteria included semantic precision, an intuitive user experience, simplicity, and extensibility. To be responsive to the requirements of different DACs, data discovery and selection needed to be available at both grouped variable and individual variable levels. To support multi-modal analysis, variables derived from higher-order pre-processed data would be used to identify image derived phenotypes, genotypes, and polygenic risk scores. Machine readability was considered essential for automation, and interoperability between data models.

### Build strategy

The build strategy was to use existing tools and actual cohort data wherever possible. There are several cohort catalogues providing cohort metadata and contact details (Integrative Analysis of Longitudinal Studies of Aging – IALSA [12], The EU Joint Programme Degenerative Disease – JPND [13], The Global Alzheimer’s Association Interactive Network – GAAIN [14], European Medical Information Framework - EMIF-AD [15]. Of these, GAAIN also provides basic feasibility analysis, and EMIF-AD provides limited harmonised datasets. EMIF-AD and the Alzheimer’s Disease Data Initiative Work Bench – ADWB [16] provide facilities for federated analyses. However, none of these approaches uses a common data model. A more relevant approach is that of the Maelstrom Catalogue [17]. This proposes a four-tier data structure moving from data domains to variables. Although the top tier (data domains) is not broadly generalisable, the basic structure is convenient for data discovery and selection at levels of detail, suitable to meet the requirements of most Data Access Committees (DACs).

The C-Surv model was developed using data from four population cohorts comprising Airwave (Airwave Monitoring Study) [18], ELSA (The English Longitudinal Study of Ageing) [19], Generation Scotland [20], and UK Biobank [21], and one clinical cohort, ICICLE-PD (The Incidence of Cognitive Impairment in Cohorts with Longitudinal Evaluation-PD) []. These studies provided a breadth of data by which the feasibility of developing a comprehensive and yet user-friendly data model could be judged. The model was developed iteratively, being expanded and revised for consistency cohort by cohort. The model was then used to fully curate all the data available to DPUK from the Airwave, ELSA (derived variable dataset), ICICLE-PD and Generation Scotland cohorts.

### Use case 1: Data Discovery

To explore the potential for C-Surv to support data discovery, it was used to develop the DPUK Cohort Explorer feasibility tool. Cohort Explorer is designed to allow users to establish the availability of data i.e. the number of participants with data, according to variable, across cohorts, prior to making a data access request. It enables users to avoid requesting combinations of variables that collectively have high levels of missingness.

Assessing feasibility in a multi-cohort environment requires the harmonisation of data across datasets. Harmonisation (the equivalence of values and/or distributions for variables across datasets) goes beyond the conventions of a common data model. However, a common data model provides a context for evaluating the suitability of variables for harmonisation. C-Surv was applied to 11 collaborating DPUK cohorts (n = 123,554).

### Use-case 2: Data Processing

As part of an ongoing analysis of life stress and mental wellbeing during SARS-Cov-2, comparison was made between preliminary data processing using native cohort data (that provided by the cohort) and C-Surv curated data. For a core dataset of 25 variables, the time required for two UK cohorts (ELSA, Generation Scotland) to discover native data and prepare it for analysis, was compared to the time required to discover C-Surv curated data and prepare it for analysis.

## Results

### Landscape review

Data structures vary considerably across cohort datasets, reflecting the conceptual frameworks of the original investigators. Variable labelling conventions were largely project-specific, and whilst suitable for in-house analysts, might be opaque to third-party users. Documentation varied considerably with no widely used structure or content. Data selection and access request procedures also varied considerably. Some DACs require individual variable selection, whilst others allow the selection of pre-defined groups of variables e.g. all cognitive variables. A small number of DACs allow virtually complete datasets to be accessed. These approaches to data selection represent compromises between administrative convenience and the articulation of scientific rigour.

### Stakeholder engagement

The two stakeholder workshop involved 10 and 9 participants respectively, of which two participants also represented DPUK. All stakeholders recognised the utility of a common data model, although reservations were expressed as to whether this was possible given the complexity of cohort datasets. Developing a comprehensive taxonomy for research phenotypes was seen as a separate problem from providing tools for data discovery. Although the prospect of rapid data discovery was universally welcomed, doubt was expressed as to the value of superficial data discovery tools that provide little information on distributions and missingness. This information was seen as essential for preparing informed and targeted data access requests. Cohort site-visits and public presentations of C-Surv provided little further information and did not generate improved solutions.

From the landscape review and the user consultation it was concluded that there was strategic value in the development of a common data model for cohort data and to focus the development of C-Surv on supporting data discovery and selection, and using this as a basis for the development of more powerful data discovery tools.

### Model development

**Fig. 1:**
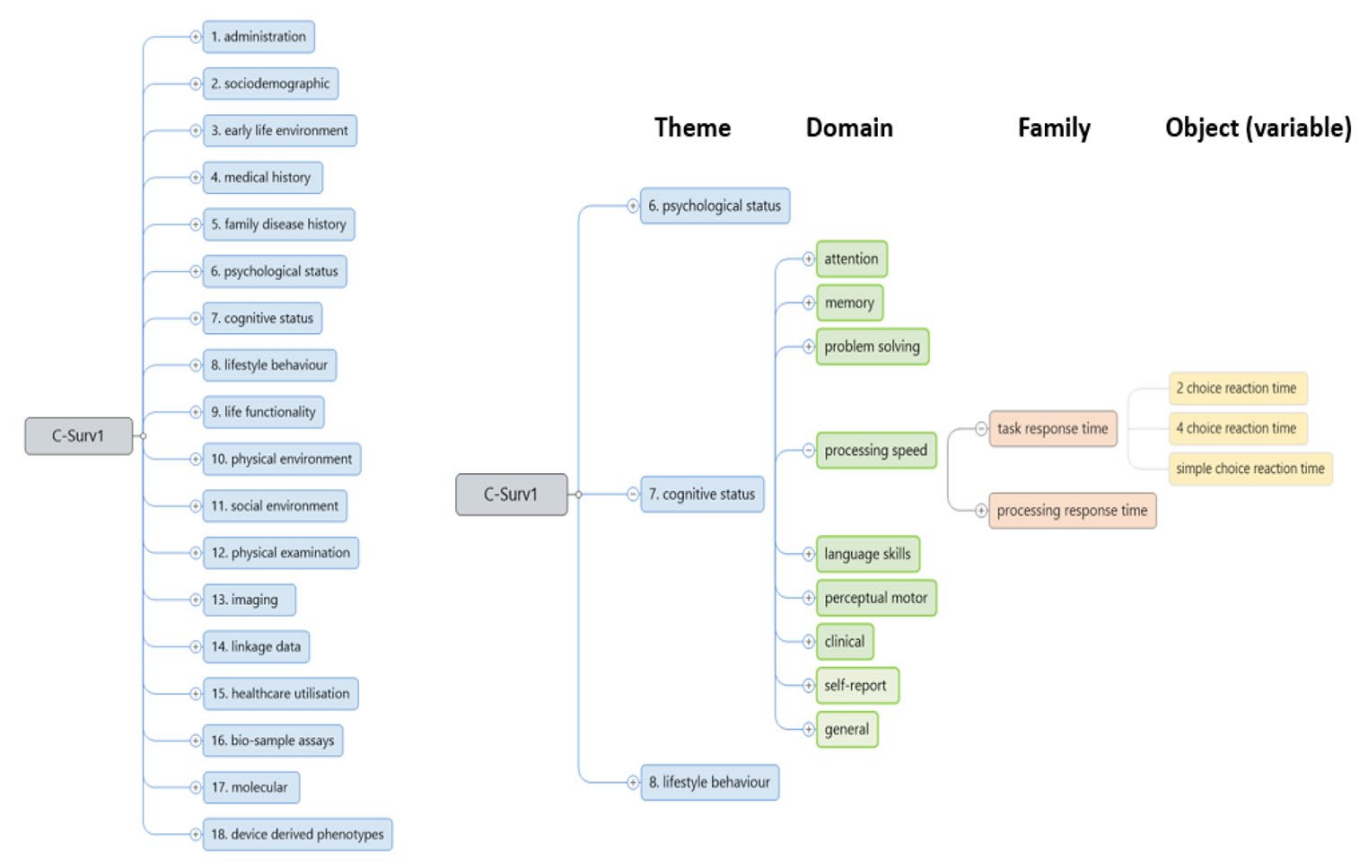
Schematic of the C-Surv data model

The iterative application of the model to data from Airwave, ELSA, Generation Scotland, ICICLE-PD, and UK Biobank found that these datasets could be organised into a relatively small number (n = 18) of ‘themes’ describing common usage and/or data modality (Fig. 1). For example, ‘Cognitive Status’ (theme 7) describes a user defined area of interest whilst the ‘Imaging’ (theme 13) describes a technology driven data modality. These themes provided the basic organising principle for developing the ontology.

### Ontological design

Following Maelstrom, the design adopted is a simple four level acyclic taxonomy intended to capture the breadth of data typically collected in research cohorts. This tiered structure supports grouped and individual variable selection. Class membership and naming at levels one to three were pragmatic decisions based on DPUK Data Portal user behaviour and the desire to maintain a four level structure for tool development purposes. Level 4 described the data object i.e. the measured variable. At this level naming was designed to uniquely identify the variable in the context of a longitudinal study.

### Data model structure

C-Surv comprises 18 data themes (level 1) leading to > 146 data ‘domains’ (level 2), > 500 data ‘families’ (level 3) and then to a growing number of data ‘objects’ (level 4). Typically data objects are variable level observations, or in the case of complex measures, such as psychometric test scores (Fig. 1). To the extent that evidence was available from DPUK access requests, the organisation of each level reflected the types of variable requests that are more frequently made. For example, typically a request would be made for all processing speed variables, rather than just choice reaction time, and so processing speed was used as a domain category. The administrative data theme includes selective metadata. More detailed metadata can be found at: Cohort Directory - DPUK Data Portal (portal.dementiasplatform.uk/CohortDirectory)

### Variable Naming

Key to utility is an informative ‘object’ (variable) name. Objects are defined pragmatically as the level of measurement used in most analyses. The object name is a complex proposition with 4 elements comprising cohort, measurement, serialisation (repeated measurement within a single data capture period), and study wave (repeated measurement between data capture episodes). These elements are considered to be the minimum required to uniquely and conveniently identify an object in dataspace. An example object name is given below:

### Gen_painchestevr_0_1

The cohort is identified using a three-digit alphabetic character (GEN for Generation Scotland). The measurement is described by an alphanumeric abbreviation (PAINCHESTEVR for: ‘Do you ever get pain or discomfort in your chest?’). This is followed by an integer representing the location of the variable within a sequence of repeat measurements within a study wave (_0 indicates there were no repeat measurements). Finally, an integer suffix indicates study wave (_1 for recruitment, _2 for first follow-up, etc.).

For survey data the measurement abbreviation is limited to 12 characters. For imaging, omics, and device data it is limited to 17 characters. Where questionnaire item level measurement is relevant, q# is added to the object name. For example, GEN_SPQq1_0_1 is an item from Generation Scotland (GEN) within the Psychological Status category (category 10), from the Schizotypal Personality Questionnaire (SPQ), question 1 (q1), administered with no repeat measurement in wave 1.

Abbreviations are selected to reflect the meaning of the full variable name used in data capture. They are upper-case, syllable based, using word fragments as abbreviations and numeric characters to facilitate easy interpretation. Consistency of abbreviations is maintained where possible. Constants are lower case for example, just as ‘q’ is used to represent question (or item), ‘r’ is used to represent range and ‘d’ is used to represent a decimal point. For example, AVG08H00r08H59 is an item from accelerometry data (average acceleration between 08h00 and 08h59). The intention is for the tiered structure and variable name to efficiently direct attention; providing sufficient information for analysts to consider which data are relevant. However, a constrained variable name is unable to capture the full context of a measurement; the same test or construct may be assessed differently between studies. Neither can a variable name fully anticipate future uses. Our view is that whether or not sufficient complexity is captured in the variable name, it is helpful for the variable to be more fully annotated by users in the data dictionary. The preparation of standard data dictionaries for DPUK datasets will follow their curation to C-Surv, and will be available online.

### Value labelling conventions

To provide correspondence between native data (that transferred to the Data Portal by data controllers) and curated data, native data value labels are retained. However, for widely used measures, value labels are standardised using common conventions. For example, missing is scored ‘.’ following the Stata [23] convention, gender is scored ‘2’ for female and ‘1’ for male. For several widely used measures imperial scaling is converted to metric. For example, height is recorded in centimetres and weight in kilograms. In C-Surv the missing indicator is reserved for an absence of recorded data as indicated by the cohort. Categories such as “prefer not to answer” and “don’t know” are coded as values. This preserves information, allowing inclusion/exclusion decisions to be made per hypothesis. For variables that can be either self-reported or formally diagnosed, the suffix ‘DX’ is added to the variable name. For example, self-report PTSD is coded PTSD and ICD-11 diagnosed PTSD is coded PTSDDX.

### Use case 1: Data Discovery

**Fig. 2:**
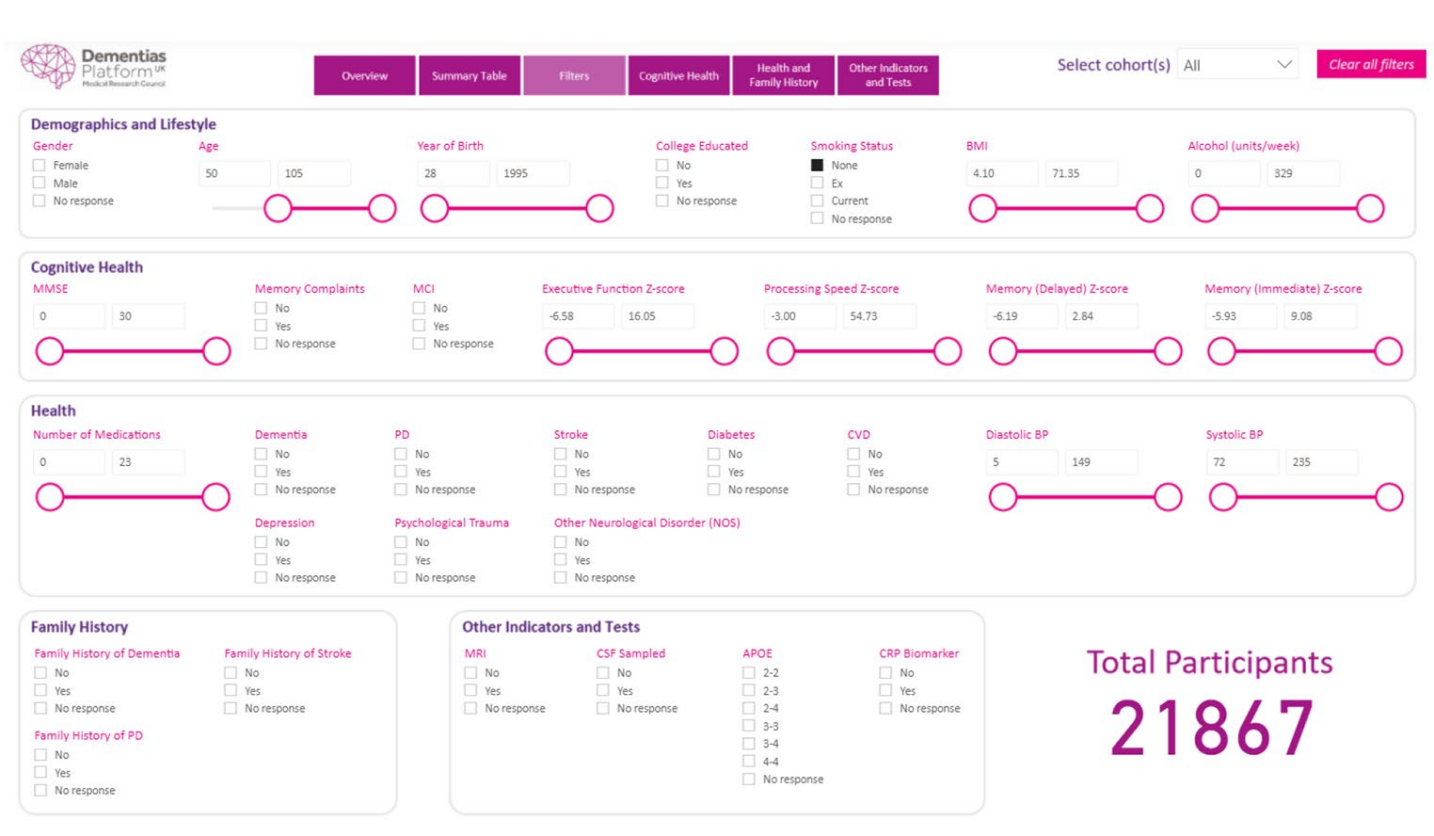
Cohort Explorer screen shot

Whilst C-Surv has been developed using all the data available to DPUK from the five collaborating cohorts, a subset of 32 variables was harmonised to inform the design and to populate the Cohort Explorer data discovery tool (Table 1). The selection of variables reflects the frequency of variables requested in dementia focussed DPUK data access applications. These variables represent a wide range of modalities and formats including imaging, genetic, and survey data. The number of variables reflects the limitations of the visualisation tool. The tool provides an interactive dashboard allowing users to select cohorts, variables and value ranges of interest. For example, of the 123,554 members of the 11 cohorts, 57,499 are aged 50 + and of these 21,867 are lifetime non-smokers (Fig. 2). However, if APOE4 status (homozygous or heterozygous) is added the numbers drop to 1,666. This is critical information when planning an analysis.

**Table 1:** Harmonised variables available in Cohort Explorer

Theme	Domain	Family	Object label
2. Sociodemographic	Demographic indicators	Age	Year of birth
			Age
		Gender	Sex
	Education	Educational experience	Years education
4. Medical history	Nervous system	Chronic neurological disorders	Dementia Diagnosis
			PD Diagnosis
		Episodic disorders	Other neurological disorders
	Circulatory	Cardiovascular disorders	CVD
			Stroke
	Self-report medical history	General health	MCI
		Medications self-report	Prescription medications
5. Family disease history	Nervous system	Chronic neurological disorder Family member	Family history dementia
			Family history PD
	Circulatory	Cardiovascular	Family history stroke
6. Psychological status	Self-report mental health	Depression	Depression scale
		Trauma	PTSD
7. Cognitive status	Memory	Short term/working memory	Immediate recall
		Long term	Delayed recall
	Problem solving	Planning	Executive function task
	Processing speed	Task response time	Reaction time task
	Self-report	Memory	Subjective memory complaint
		Cognition	Cognitive impairment
8. Lifestyle behaviour	Substance use	Alcohol	Alcohol units/wk
		Tobacco	Smoking status
12. Physical examination	Musculo-skeletal	Structural	BMI
	Circulatory	Cardiovascular	BP systolic
			BP Diastolic
13. Imaging	Brain	MRI	MRI images
16. Bio-sample assays	Blood	Haematology	CRP
	CSF	Proteins	CSF Tau
17. Molecular	Genomics	SNP	APOE

### Use case 2: Data Processing

For the life stress and wellbeing analysis, using data from ELSA and Generation Scotland, a core data set of 25 variables was identified and prepared for analysis. This involved searching the data catalogue, selecting variables, and writing code to translate the variable naming and value labelling formats to the preferred conventions of the analyst. This had to be repeated for each study, and required 5–6 h per cohort. Using C-Surv curated data, discovery required 30 min for both cohorts, and a further hour for the C-Surv curated data to be translated into the analyst’s preferred conventions (the same code being applicable to both cohorts). The results of this analysis will be reported separately.

## Discussion

Following a landscape review, user consultation, and using data from five collaborating cohorts, the C-Surv data model was developed to investigate the utility of a common data model for research cohort data. The data model, optimised for data discovery and variable selection, was used to develop the Cohort Explorer analysis feasibility tool.

## Advantages

The C-Surv structure and variable naming conventions were able to accommodate all the data types and formats found in the native cohort data. These included survey, imaging, genetic, and environmental data. The combination of user-based and technology-based groupings, including the coding of medical history using ICD-11, was pragmatic; nosological significance was not intended. That a four-level nested hierarchy can be applied to diverse datatypes is unsurprising. The challenge is to apply the hierarchy in a way that is useful. An example of utility was shown in providing an ontological framework necessary for the development of a multi-cohort data discovery and variable selection tool. A second use-case demonstrated that substantial time savings are obtained from using data curated to a common data model. These findings suggest that a common data model is useful for data discovery, variable selection, and analysis. These benefits apply to a range of observational designs including cohorts, case-control studies, and research registers.

## Limitations and future directions

The test of the model was not comprehensive. It was not applied to electronic health record data, or device generated data such as accelerometry. The curation strategy is that these and other complex data such as imaging and genetics require pre-processing before curation to C-Surv. For example, whilst C-Surv can be used for imaging derived phenotype discovery, this is dependent on prior derivation of those phenotypes.

Cohort data are dynamic with many cohorts being active with further data collection and C-Surv will evolve to reflect this. However, updating a data model and updating datasets is less demanding that the initial curation, and continuity can be maintained through version control. This illustrates that ontologies and their attendant data models are purpose-specific. C-Surv is optimised for multi-modal end-user data discovery and selection. This is in contrast to models designed to establish common metadata standards for genetic research cohorts, such as the Genomics Cohorts Knowledge Ontology - GECKO model of the CINECA (Common Infrastructure for National Cohorts in Europe, Canada, and Africa) consortium [24], or models designed to follow the flow of data collection such as that used in UK Biobank [21].

Manual curation is labour intensive, and vulnerable to error. Maintaining quality control is an important issue and systems guaranteeing the provenance of curated datasets are required for the confidence of the community to be retained. Preliminary attempts at automation, using supervised machine learning, have achieved correct curation of around 70% of variables. Although improved performance may be anticipated, it is unlikely that 100% accuracy can be achieved reliably. Inability to achieve full automation raises the issue of quality control. Uncontrolled use of a data model risks undermining its scientific value as its standards are unlikely to be applied consistently.

The utility of Cohort Explorer was constrained by the dashboard being limited to the visualisation of 32 variables. Re-designing the dashboard to increase the number of variables would improve the value of the tool. Cohort Explorer is also limited to identifying the amount of data available according to cohort and variable combination. Whilst this is important, the addition of a power calculator and some preliminary regression analytic capability would add value. It is likely that a persistent and widely used data model would incentivise commercial development of more powerful data discovery tools. A further limitation in Cohort Explorer is the methods used for harmonisation. Data harmonisation is implicitly purpose-specific and may vary according to hypothesis and analytic strategy. However, for the purpose of data discovery, the relatively simple strategies used here of standardising scale values and, where appropriate, transforming to standardised distributions are likely to be sufficient. Cohort Explorer can be found at (https://portal.dementiasplatform.uk/CohortExplorer). As the tool uses individual-level cohort data it requires a DPUK account to access. This can be obtained upon application to: https://portal.dementiasplatform.uk/Account/Register .

Although the benefits of common data models for cohort data are clear, models have been slow to develop. This is likely due to the substantial development cost and the uncertain benefit accruing to the developer. Also, curating data specifically for the benefit of third-party researchers is a recent phenomenon and individual research teams are rarely resourced to curate data beyond their own needs. Cost-related barriers may be addressed by curation services. In this scenario, data producers have their data curated to one or more common standards by third-parties specifically resourced for this purpose. In the experience of DPUK this is a convenient and cost-effective solution for cohort research teams. It is also cost-effective for the community more widely, as once a processing pipeline has been established, cost per datum reduces with each application.

A further barrier to uptake is achieving consensus within the scientific and potentially clinical communities, as a data standard is only useful to the extent it is adopted. However, science needs to start somewhere, and only by developing data standards and using them will the ‘cream rise to the top’.

In addressing these barriers, it is helpful to make a distinction between a common data model and harmonised datasets. The goal of a common data model is to standardise data structures and naming conventions across datasets. In contrast, the goal of harmonisation is to achieve inferential equivalence across datasets. For example, are two variables in different datasets measuring the same latent construct? The answer to this question is independent of the data model(s) used. Although several harmonisation initiatives are ongoing such as CLOSER [25], and Dementia Platform Korea [26], here we are concerned with enabling individual datasets to be research-ready.

## Conclusion

A common data model, used to prepare data to a defined standard for research readiness, offers many advantages. These advantages will accrue as data grow in complexity, scale, and sensitivity. Here we demonstrate the feasibility and utility of applying a common data model to research cohort data.

However, this is also an attempt to stimulate and contribute to a wider debate on how to provide wide access to research-ready data at scale and speed. Building and maturing a data model is a collaborative and iterative process. It requires the engagement of the user community, particularly those in lower resource settings, for benefit to be widely realised. DPUK is collaborating with Dementias Platform Australia (DPAU) (https://www.dementiasplatform.com.au/) and The Alzheimer’s Disease Data Initiative (ADDI) (https://www.alzheimersdata.org/ad-workbench) to apply C-Surv to international datasets. DPUK welcomes further collaboration in the development of C-Surv, and other tools and technologies that enable access to research-ready data at scale and speed.

## Electronic supplementary material

Below is the link to the electronic supplementary material.

Supplementary Material 1

## Data Availability

Not applicable.
